# Cardiovascular and Muscular Consequences of Work-Matched Interval-Type of Concentric and Eccentric Pedaling Exercise on a Soft Robot

**DOI:** 10.3389/fphys.2017.00640

**Published:** 2017-08-31

**Authors:** Martin Flück, Rebekka Bosshard, Max Lungarella

**Affiliations:** ^1^Department of Orthopedics, University of Zurich Zurich, Switzerland; ^2^Zurich Center for Integrative Human Physiology, University of Zurich Zurich, Switzerland; ^3^Department of Health Sciences and Technology, ETH Zurich Zurich, Switzerland; ^4^Dynamic Devices Zurich, Switzerland

**Keywords:** muscle, eccentric, concentric, respiration, lactate, robot, power, feedback

## Abstract

Eccentric types of endurance exercise are an acknowledged alternative to conventional concentric types of exercise rehabilitation for the cardiac patient, because they reduce cardiorespiratory strain due to a lower metabolic cost of producing an equivalent mechanical output. The former contention has not been tested in a power- and work-matched situation of interval-type exercise under identical conditions because concentric and eccentric types of exercise pose specific demands on the exercise machinery, which are not fulfilled in current practice. Here we tested cardiovascular and muscular consequences of work-matched interval-type of leg exercise (target workload of 15 sets of 1-min bipedal cycles of knee extension and flexion at 30 rpm with 17% of maximal concentric power) on a soft robotic device in healthy subjects by concomitantly monitoring respiration, blood glucose and lactate, and power during exercise and recovery. We hypothesized that interval-type of eccentric exercise lowers strain on glucose-related aerobic metabolism compared to work-matched concentric exercise, and reduces cardiorespiratory strain to levels being acceptable for the cardiac patient. Eight physically active male subjects (24.0 years, 74.7 kg, 3.4 L O2 min^−1^), which power and endurance performance was extensively characterized, completed the study, finalizing 12 sets on average. Average performance was similar during concentric and eccentric exercise (*p* = 0.75) but lower than during constant load endurance exercise on a cycle ergometer at 75% of peak aerobic power output (126 vs. 188 Watt) that is recommended for improving endurance capacity. Peak oxygen uptake (−17%), peak ventilation (−23%), peak cardiac output (−16%), and blood lactate (−37%) during soft robotic exercise were lower during eccentric than concentric exercise. Glucose was 8% increased after eccentric exercise when peak RER was 12% lower than during concentric exercise. Muscle power and RFD were similarly reduced after eccentric and concentric exercise. The results highlight that the deployed interval-type of eccentric leg exercise reduces metabolic strain of the cardiovasculature and muscle compared to concentric exercise, to recommended levels for cardio-rehabilitation (i.e., 50–70% of peak heart rate). Increases in blood glucose concentration indicate that resistance to contraction-induced glucose uptake after the deployed eccentric protocol is unrelated to muscle fatigue.

## Introduction

External work being produced during muscle activation, and its metabolic cost, depend on whether the resulting contraction leads to shortening or lengthening of the muscle-tendon-bone unit (Jones et al., 2004). Already in 1925, Sir A.V. Hill demonstrated that lengthening type contractions produce more force and power than shortening (or isometric) type of contractions (Padulo et al., 2013). Later it was shown that the metabolic cost of the eccentric type of exercise is lower than with the concentric type of exercise (Bigland-Ritchie and Woods, 1976; Isner-Horobeti et al., 2013). This is reflected by a reduced rise in blood lactate concentration after short intense, or endurance type, eccentric leg exercise compared to work-matched concentric exercise (Horstmann et al., 2001; Penailillo et al., 2013). The underlying processes rendering eccentric contractions more efficient, involve changes in the energetics of the cross-bridge cycle, the retrieval of elastically stored energy within the muscle-tendon unit and neuronal mechanisms (Ryschon et al., 1997; Isner-Horobeti et al., 2013; Nishikawa, 2016).

Eccentric types of endurance exercise are attractive for subjects, wishing to specifically strengthen skeletal muscle's force and fatigue resistance respective to cardiovascular aspects of exercise. Due to the reduced metabolic and cardiovascular cost at a same mechanical power than concentric exercise, eccentric types of endurance exercise are especially attractive for subjects with cardiovascular limitations such as patients with cardiac disease or chronic obstructive pulmonary disease (COPD; (Steiner et al., 2004; Isner-Horobeti et al., 2013; Philippe et al., 2016). During rehabilitation it is recommended for these patient groups to perform at a lower relative percentage of maximal heart rate than healthy subjects, i.e., 50–70% compared to 70–85% of maximal heart rate (Carre, 2010; Mampuya, 2012). In this regard integrating eccentric exercise into a protocol with high intensity intervals (Helgerud et al., 2007) may be of particular interest because this provides a potent stimulus for muscle adaptation at a lower metabolic load and reduced net exercise time (Fu et al., 2014). However, eccentric types of contraction lead to more wear-and-tear of the implicated muscle structures, which may damage muscle fibers, reduce power output, and affect glucose uptake (Asp et al., 1995; Cook et al., 2015). To our understanding the respective mechanical and metabolic characteristics of interval-type eccentric exercise has not been evaluated in a fully matched situation of interval-type exercise (Abbott et al., 1952; Bonde-Petersen et al., 1972; Isner-Horobeti et al., 2013). Specifically, this concerns information on the contribution of concentric and eccentric forms of contraction to the production of mechanical output in the extension and flexion phase of the exercise work cycle. This lack of knowledge is related to the fact that the controlled imposition of workload during comparable concentric and eccentric exercise, and real-time monitoring of the effective mechanical output and oxygen consumption, poses specific demands on the exercise machinery and analytic equipment.

Here we compared the cardiorespiratory and muscular consequences of work-matched eccentric and concentric types of two-legged exercise in physically active, healthy subjects. In the perspective of applying the protocols to exercise rehabilitation of patients with cardiovascular limitations, we adopted an interval-type exercise because this allows for intermittent recovery between the imposed cycles of work (Kavanagh and Shephard, 1975). We deployed a soft robotic device with haptic feedback (Allegro^TM^, Dynamic Devices), which met the criteria for a clinical investigation, and which developed distinct advantages regarding robustness, accuracy, and versatility respective to the eccentric cycle ergometer, which we characterized previously but which was not any longer an option (Zoll et al., 2006; Mueller et al., 2011). Specifically, the soft robot allowed to impose protocols of knee extension and flexion that were fully adjustable regarding force and speed in the concentric and eccentric phase of loading, through pneumatically operated independent pedals. The device also allowed to resolve the contribution of concentric and eccentric work to mechanical output at a high accuracy and temporal resolution, and separately for both legs, and permitted the quantification of peak power, all of which was fully traceably though software enabled storage and retrieval of the data.

We hypothesized that the eccentric interval-type of exercise lowers cardiorespiratory strain and strain on glucose metabolism compared to the work-matched concentric exercise, similarly as previously reported for continuous forms of cycling exercise (Kavanagh and Shephard, 1975; Bigland-Ritchie and Woods, 1976). Finally, we examined whether the workload and intensity of the established interval-type of exercise is sustainable for physically active subjects and within the recommendations for cardiac patients.

## Materials and methods

### Subjects

The Ethics commission of the Canton of Zurich, Switzerland, approved the study protocol. Healthy, physically active and non-smoking young male subjects were recruited from the canton of Zurich via word of mouth and advertisements at the University and Swiss Federal Institute of Technology (ETH) of Zurich. Prior to the start of the study, the study participants were informed orally and signed a written consent. Fifteen subjects volunteered for the study and eight subjects reported to the laboratory on all test days to conduct the exercise protocols. Three of the 15 subjects dropped out after the baseline test because of injuries during their leisure activities. Four other subjects resigned because of management problems due to a time lag between the recruitment and entry into the protocol.

### Design

**Figure 1:**
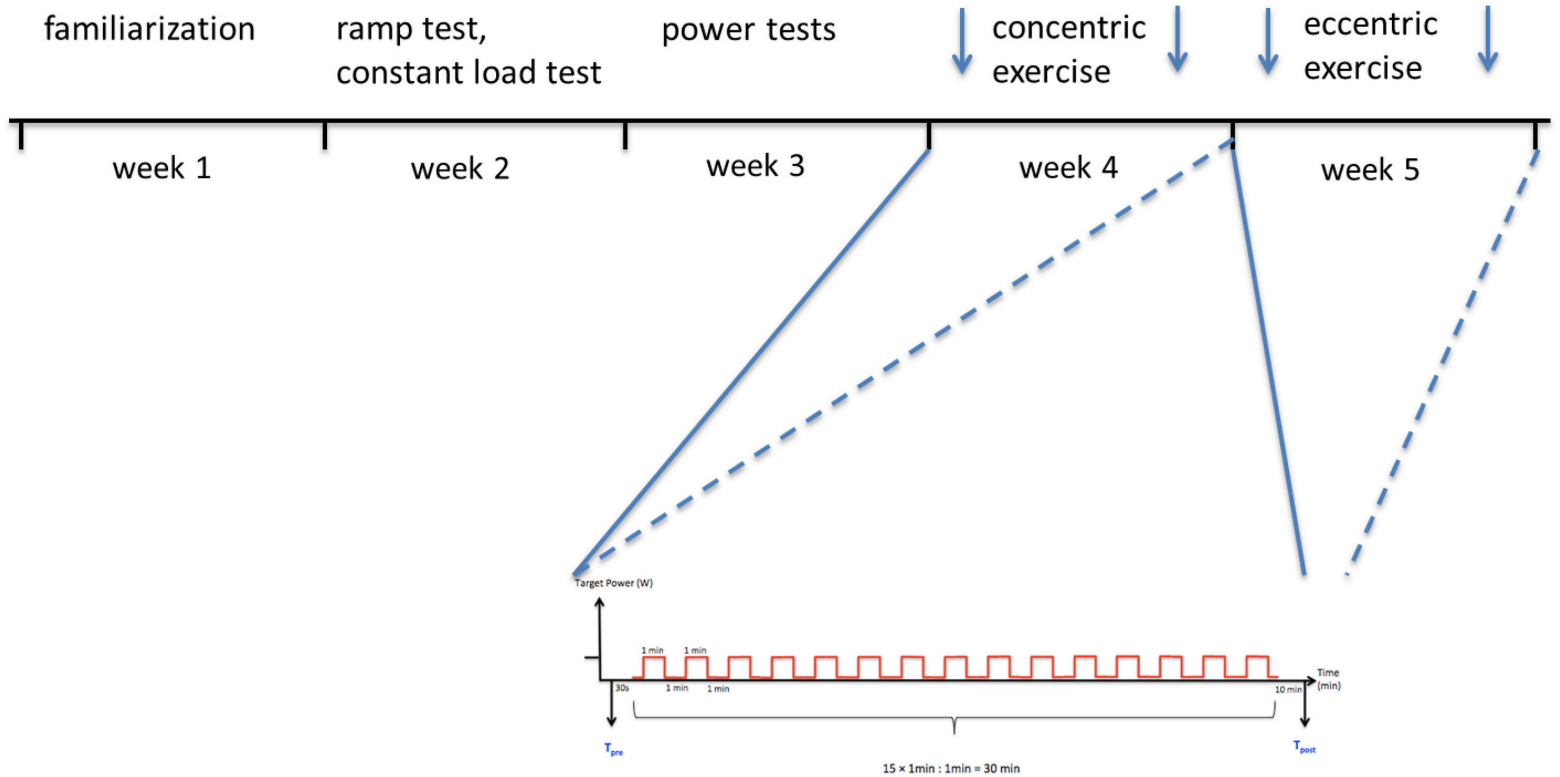
Sketch summarizing the study protocol. Subjects conducted a number of tests over 5 weeks with ample time to allow recovery from the individual types of exercise. In the first weeks this included a familiarization to the equipment. In the second week subjects performed a ramp test (to determine maximal cardiovascular performance) and an endurance exercise at constant load (to determine the time-to-exhaustion). In the third week the subjects carried out power tests on the soft robot or force plate to determine maximal force and power of single knee extensions. In the fourth and fifth week, subjects carried out a session of interval-type concentric exercise (week 4), and eccentric exercise (week 5), on the soft robot with the same overall work output for both legs. Thereby force and blood serum measurements were made before and after the exercise sessions (arrows). Below, scheme summarizing the 15 sets of 1-min exercise, during which 30 contractions (work cycles) were performed with each leg, and the subsequent periods of recovery.

The entire protocol comprised five appointments, which were interspersed over a period of 5 weeks (Figure 1). It served to properly characterize the subject's fitness and to assess the consequences of power-matched interval-type of eccentric and concentric exercise on a soft robot on cardiorespiratory function and selected metabolic parameters during exercise and mechanical output post-exercise. Intensity of interval-type exercise was selected based on the physical performance of the subjects in power tests on the soft robot.

In the first 2 weeks of enrolment, subjects familiarized to the equipment and their anthropometry was assessed. Subsequently, and on separate days, the subjects underwent a ramp test and endurance exercise at constant load at separate days on a cycle ergometer to determine Ppeak and maximal cardiovascular performance, and time-to-exhaustion, during endurance-type leg exercise, respectively. In the third week subjects carried out power tests on the soft robot and performed jumping mechanography to determine maximal force and power of single knee extensions. In the fourth and fifth week, subjects carried out a session of interval-type concentric exercise (week 4), and eccentric exercise (week 5), on the soft robot with the same overall work output for both legs. The sustainable intensity of the first exercise in concentric modality served to set, and match, the intensity for the eccentric exercise. Mechanical output and cardiovascular parameters were determined during the exercise sessions. Force, power and blood metabolites (glucose and lactate) were assessed before and after the exercise sessions, with lactate also being assessed during exercise. Participants were not allowed to do strenuous leg exercise 48 h before each appointment and were instructed to keep their general training in the rest of the week and maintain their usual nutrition without consumption of coffee and alcohol 24 h before the test date.

### Description of the soft robotic device

The Allegro soft robot (Dynamic Devices, Zurich, Switzerland, http://www.dynamicdevices.ch) is a closed kinetic chain, force-controlled interactive training, and testing device for the lower extremities. It features low-latency multimodal (visual, auditory, and haptic) feedback (<30 ms), intrinsically compliant pneumatic actuation, forces and speeds matching those of humans [rate of force development (RFD) > 15 kN s^−1^], and a wide range of online sensory–motor testing and measuring tools. The Allegro device has a leg press layout and consists of two independent pedals and free footplates. The pedals are directly and rigidly connected to a force generating linear actuation mechanism through shafts and levers. The seat has an adjustable height and a declinable backrest.

At the core of the Allegro's actuation mechanism are pneumatic artificial muscles (PAMs). These are tube-like actuators characterized by a smooth, accurate, and fast response that can produce a pulling force while contracting. The force exerted by the PAM when pressurized with air is a function of its internal chamber pressure and its length. For each device, a high precision factory-calibrated load cell is used to model the non-linear relationship between force, pressure, and PAM length.

The Allegro calculates and controls the force exerted by the PAM (accuracy: 2% full scale), its contraction speed and hence also the external mechanical power applied to the user. Visual feedback of the measured performance through a work cycle is provided via a monitor. The Allegro is equipped with several fundamental test protocols allowing to asses motor accuracy, peak force, positive impulse, negative impulse, net impulse, and power for cyclic movements, reaction time, force control, and force steadiness.

### Ramp test

Ppeak and cardiovascular parameters (peak oxygen uptake) of repeated knee extensions, were determined on a cycle ergometer (Cardioline xr100, Ergoline, Bitz, Germany) with an incremental protocol to exhaustion using ergospirometry with an Innocor Device (Innovision, Glamsbjerg, Denmark). Cardiac output was measured using the inert gas rebreathing method. Subjects were equipped with a facemask (Hans Rudolph, Shawnee, KS, USA) and a bacterial filter (PALL PF-30S, Innovision, Glamsbjerg, Denmark), which were connected to the Innocor machine. Expired carbon dioxide, oxygen concentration, and airflow were measured breath-by-breath and used to calculate oxygen uptake and ventilation. The test started always with a measurement at rest in sitting position: Subjects practiced rebreathing in a closed system with a 3–4 L volume bag and an infrared photo acoustic gas analyzer. They had to comply to a breathing frequency of 20 min^−1^, for 6 repeated inspiration and respiration maneuvers. Sulfur hexafluoride (SF6) and nitrous oxide (N_2_O), which were diluted with atmospheric air, were used as rebreathing gases. Immediately after the rebreathing, the exercise began and the measurements started. The initial load of the exercise protocol was 100 W. Target load was increased by 20 W every second minute under a freely chosen pedaling rate between 70 and 100 rpm until voluntary exhaustion occurred. Tests were aborted when the subject stopped pedaling or when the frequency felt below 70 rpm. The corresponding Ppeak was recorded. The measurement of the cardiac output was repeated at volitional exhaustion to measure the corresponding peak value. Heart rate was measured at rest, every 2 s during exercise, at volitional exhaustion and 8-min after exercise with a Polar Sensor (Polar FT4, Polar Electro).

### Endurance exercise at constant load

Time-to-exhaustion was determined in a constant load test on the cycle ergometer (Cardioline xr100, Ergoline, Bitz, Germany). The load was set to 75% of the Ppeak from the ramp test in line with recommendations (Pollock, 1973). Subjects cycled at a pedaling frequency between 70 and 100 rpm. The test was aborted when the frequency could not be held anymore.

### Power tests

Maximal values of real, reactive and negative force, and power of single knee extensions were assessed with specific tests on the soft robotic device (Allegro, Dynamic Devices; Supplementary Figure 1). For further details, the reader is referred to animated illustrations: https://www.youtube.com/watch?v=EJ34D0btS6k; https://www.youtube.com/watch?v=nAULzLl5zyE. The tests on the Allegro device were part of the default settings of the robot. They were carried out in a seated position by applying force to two decoupled pedals, which were controlled by the action of the PAMs (see above). Before the tests, the chair of the machinery was adjusted to allow an optimal positioning and movement ranging from 90° knee flexion and full extension while subjects were sitting on the robot and positioning the feet on the two pedals. All maneuvers were carried out under supervision and instructed with a monitor, which displayed the respective commands as well as the target and effective mechanical output in real-time.

Each test contained three repetitions (trials) with a 10 s break in between. Immediately after these three trials the next test started. The tests were carried out in the following order real power test, reactive power test and negative power test. For the *real power test*, each leg was charged with a load corresponding to half of the body weight at a flexion angle of 90°. Upon the appearance of a signal, the pedals were pushed as hard as possible to full leg extension. After 10 s of rest the next trial began. The *reactive power test* was carried out in a stretched position (knee angle of 10°) at the same load as the real power test. During this test, the legs were flexed and extended as quickly and powerful as possible. The reversal point of the knee was at a knee angle of 85–95°. The recovery time was 10 s in between trials. For the *negative power test* the load was set to 75% of the body mass per leg. Legs had to be brought to a knee angle of 70°, which position was displayed with a green line on the screen in front of the subjects. The aim of the maneuver was to hold the position and damp the rebound of the machine upon the application of the load as quickly as possible. Again 10 s rest was allowed between trials.

Jumping mechanography was carried out with a ground reaction force plate (Leonardo Mechanograph®, Novotec, Pforzheim, Germany) being connected to a desktop computer using an integrated analog digital board and software system (Leonardo Mechanography GRFP version 4.2, Novotec, Pforzheim, Germany). The platform had two symmetrical force plates and held eight force sensors, which measured the ground reaction force exerted on the plate. Counter-movement-jumps (CMJs) assessed reactive power with a phase-shifted velocity and force by 90°. Squat-jumps (SJs) assessed real power with an in-phase force and velocity. Three vertical two-legged CMJs, being separated by 30 s of rest were carried out, and followed by three SJs. Subjects stood without shoes, feet in shoulder length apart, on the plate. During the CMJ the arms were hanging loosely at their sides and moved freely during the jump. The goal was to jump as high as possible and stand still again after landing to end the measurement. SJs were carried out with the hands holding on the hips for the whole maneuver. Upon initiation of the signal, subjects had to flex their knee joint down to an angle of 90–100°, and hold the position for a second before subsequently jumping as high as possible. Additionally, single two-legged jumps and three one-legged multiple jumps (hopping) were performed on the force plate to determine Ppeak, force, and stiffness (Mueller et al., 2015). For this procedure, the subject performed with the dominant leg and began to jump only on the forefoot with a stiff knee as fast as possible. Upon a verbal command, subjects were asked to jump as high as possible with stiff knee and without touching the ground with the heel. Any jumps with heel contact were excluded from the analysis.

### Concentric exercise and eccentric exercise on the soft robot

Interval-type of exercise sessions were carried out under standardized conditions on the Allegro soft robot using a protocol which simulated the pedaling exercise during bicycling (Dynamic Devices, Zurich, Switzerland). One parameter setting in the protocol allowed switching between a mostly concentric type and a mostly eccentric type pedaling action. Subjects were asked to refrain from strenuous exercise in alcohol in the 48-h before the exercise. Subjects continued their normal training and nutrition but were asked to stop the consumption of coffee during the test phase. The exercise sessions were composed of 15 sets of 1-min alternating cycles of two-leg knee extension and flexion at 30 rpm, with 1-min recovery in between (Figure 1). Exercise was carried out in concentric or eccentric modality at matched external workload corresponding to 17% of the maximal power for one leg as estimated in a real power test on the soft robot. Eccentric exercise was carried out 1 week later. Starting position was the same as described in the real power test above. Mechanical characteristics of the exercise on the soft robot were recorded at 200 Hz using high-resolution sensors. Cardiovascular characteristics (gas exchange and hemodynamic parameters) of the exercise stimulus on the soft robot were assessed using ergospirometry. Immediately before and 10-min after the exercise session power output was assessed with real, reactive, and negative power tests on the soft robot, as well as SJs and CMJs on the force plate. Capillary blood samples were drawn from a finger prick and directly applied on the measurement strip to quantify blood lactate and glucose using the Accutrend system (Accutrend® Plus System, Cobas®, Roche Diagnostics Limited, Rotkreuz, Switzerland). Glucose was assessed before exercise, immediately and 8 min after finishing the exercise. Lactate was assessed before, all 2–4 min during exercise, immediately after and 8 min after finishing the exercise.

### Data handling

Data including the parameter settings of the protocol (e.g., subject identifier, rpm, target power, work cycle number, set, time) and performance values (negative and positive force, negative and positive power, arc length and angle for each sampled time point and set for both left and right leg) were exported from the soft robotic device in the form of comma-separated files. Negative and positive work produced was calculated from the sum of positive and negative power per time increment (5 ms) for each set. As well the work performed during each work cycle was calculated using a customized Python script (Python Software Foundation) for the phases when leg muscles are expected to perform target power (i.e., during the extension phase in the concentric protocol and flexion phase in the eccentric protocol), and the “off phase” of each cycle when muscle is not expected to produce power (i.e., during flexion in the concentric protocol and extension in the eccentric protocol). For the analysis of work cycles, absolute force output was normalized to the maximal values detected in the respective set of exercise. As well the achieved power was compared respective to the target power, and differences in power between the right and left leg were calculated in relation to the average of both legs. Average values of cardiorespiratory measures (heart rate, VO_2_, RER) were calculated from the output of the Innocor measurements using MS-Excel (Microsoft, Kildare, Ireland) after aligning the measured values with the time scale.

### Statistics

Statistical analysis was carried out using SPSS (version 23, IBM). Kolmogorov-Smirnov and Shapiro-Wilks test showed that a normal distribution could not be rejected at *p* < 0.05 for any the assessed parameters pre- and post-exercise. Differences between protocols were compared with a repeated ANOVA for the repeated factor “protocol” (eccentric, concentric, constant load, ramp test). Pre- vs. post-exercise differences for physiological parameters were compared with a repeated ANOVA for the repeated factors “exercise” (post, pre) and “protocol” (eccentric, concentric). Effects were localized with the *post-hoc* test of least significant difference (SPSS, IBM). Significance was declared at a *p*-value below 5%. Effect sizes and power are shown in Supplementary Table 1. Network analysis was carried out based on linear relationships using the expression correlation network plugging of the cytoscape software (2.5.0) at a condition of |r| > 0.70. Correspondence between power and force for measurements during the real power test on the soft robot and the SJs on the force platform was assessed using Bland-Altmann analysis for mean normalized values (Giavarina, 2015).

## Results

### Subjects

The biometric and performance data of the eight subjects, which completed the study, are given in Table 1. The values of Ppeak were 3.7–4.5-times higher for the mechanographic measurements from the SJs and CMJs than the values for reactive or real power as assessed on the soft robot.

**Table 1:** Subject characteristics.

**Parameter**	**Values**
Age (years)	24.0 ± 3.5
Weight (kg)	74.7 ± 9.3
Height (cm)	181.8 ± 5.0
VO2peak (L O2 min^−1^)	3.4 ± 0.2
Ppeakramp (Watt)	250.0 ± 17.7
Ppeakreac (Watt)	1006.2 ± 119.2
Ppeakreal (Watt)	833.4 ± 103.9
Ppeakneg [Watt]	492.8 ± 115.5
Psjpeak (Watt)	3722.7 ± 612.3
Pcmjpeak (Watt)	3684.1 ± 1729.8
RFDreac (N s^−1^)	2868.1 ± 289.7
RFDreal (N s^−1^)	1920.5 ± 372.2
RFDneg (N s^−1^)	3621.1 ± 1664.7

*Mean + standard deviation of physiological parameters as assessed prior to the interval-type of concentric exercise. For the parameter names see the list of abbreviations*.

Power and force measures as determined on the soft robot were analyzed for relationships to the power values from mechanography. Maximal power (*r* = 0.79, *p* = 0.02) and force (*r* = 0.84, *p* = 0.01) as determined in the real power test on the soft robot were highly correlated with the corresponding values in the SJ test on the force plate. Mean normalized values of force and power in the SJ and real power measurements were all contained within the limits of agreement of Bland-Altmann analyses (Supplementary Figure 2). We therefore used the values for the real power, as determined on the soft robot, to set the workload of the exercise session.

### Characteristics of eccentric and concentric exercise on the soft robot

**Figure 2:**
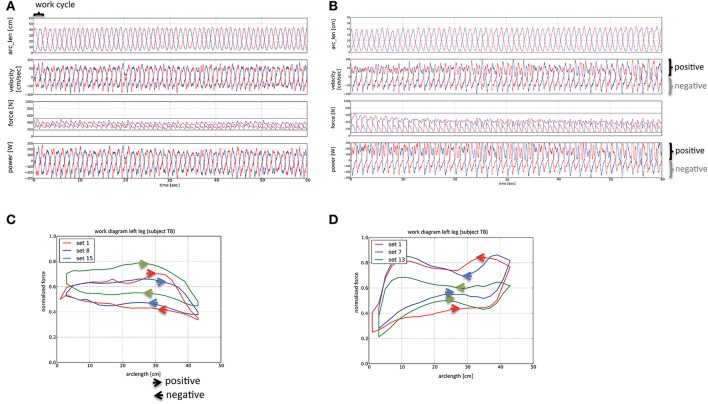
Work diagrams of the interval-types of concentric and eccentric exercise. Line graphs visualizing the sampled mechanical characteristics during 30 work cycles of each leg within one set of the concentric **(A)** and eccentric **(B)** interval exercise on the soft robot. Red and blue lines symbolize the measured parameters for the left (blue) and right leg (red). The length of a work cycle is indicated with a swung bracket (top left in panel **A**). The zones corresponding to the phases where positive and negative work is developed are indicated. Values for power, force, velocity and arc length (arc_len) are shown. Note a considerable portion of the power is produced points in the negative direction for the concentric protocol, and the positive direction for the eccentric protocol, respectively. **(C,D)** Line graphs visualizing the average of normalized force output in different sets of the interval-type of concentric **(C)** and eccentric exercise **(D)**. The direction in which work is performed is indicated with arrowheads. The inspection of the force: length relationships in **(C,D)** of the same representative subject reveals the average contribution of positive and negative force production at different arc length, and differences between sets, to the work cycle within an exercise protocol. For instance, **(C)** shows that force transits from nearly constant and higher levels in the extension phase to lower and equally constant values at the largest arc length (when legs become fully extended) in the flexion phase during the concentric interval exercise. In consequence, eccentric force is developed during the concentric protocol of interval exercise. Note that in the last cycle of this protocol the force developed during, both, the eccentric and concentric phase is higher for the displayed subject. Conversely, in **(D)** showing the work cycle during the eccentric interval exercise, one can recognize that the developed forces are higher during the flexion compared to the extension phase. Thereby the forces developed in both phases are less regular and more distinct than for the concentric exercise.

An example of the mechanical output during one set of the interval-type of exercise on the soft robot is given in the work diagram of Figure 2. On average 101 ± 2 and 107 ± 3%, respectively, of the requested performance was achieved by the subjects performing under work-matched eccentric and concentric protocols in the respective eccentric and concentric phase of the protocols. The duration of exercise was the same with the concentric and eccentric protocol.

Three subjects failed to complete the 15 sets of the interval-type of concentric exercise. The number of sets for the eccentric interval-type of exercise was consequently adjusted to allow for a matched workload. The performance values of these three subjects fell below 87% of the target workload in one leg with the concentric protocol. Over all subjects, an average of 12 sets was completed for both the concentric and eccentric protocol.

Subjects produced considerably more negative work during the eccentric than the concentric protocol, i.e., 4.7 ± 0.1 vs. 3.2 ± 0.1 kJ per set. The total work produced during the two types of soft robot exercise did not differ (*p* = 0.64) and amounted to 7.5 ± 0.1 kJ, both, per set for the concentric and eccentric protocol, respectively.

Inspection of the performance diagrams indicated that subjects also produced considerable force and power in the negative direction with the concentric protocol. On average, this amounted to 74 ± 1% of extra work. The average ratio between the developed positive to negative power was 1.2 ± 0.1 for the concentric protocol. Similarly, with the eccentric protocol there was a considerable degree of force being produced in the positive direction, i.e., an extra of 72 ± 4% of work was performed concentrically. The ratio between negative and positive power was 1.9 ± 0.1 for the eccentric protocol. An example of the force output in function of arc length during an average work cycle is given for the concentric and eccentric protocol, respectively, in Figures 2C,D. The inspection of these work diagrams shows that subjects maintained target power through a different strategy for the concentric and eccentric protocol.

For the shown example of concentric exercise, subjects maintained force at a similar level over the positive and negative direction of the work cycle, i.e., being independent of the arc length and with a rapid transition in-between (Figure 2C). By contrast with the eccentric protocol, considerable larger forces were produced in the negative compared to the positive direction of the cycle (Figure 2D).

### Mechanical effects of eccentric and concentric exercise

**Figure 3:**
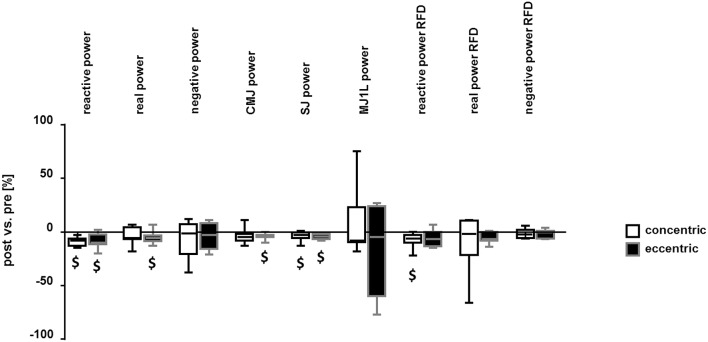
Reduction in mechanical output after interval exercise on the soft robot. Box Whisker plots visualizing the median + standard error (box and central line) and minima/maxima (whisker) of the post- vs. pre- exercise differences in Ppeak and the RFD for the concentric and eccentric protocol, respectively. Power values were assessed by the two-legged reactive power test, real power test, negative power test, CMJ, SJ, and the one-legged multiple jumps. RFD was determined for the reactive power test, real power test and negative power test. $: *p* < 0.05 vs. pre (see Table 1). Repeated ANOVA for the repeated factors of time point relative to the exercise on the soft robot (i.e., pre, post) and exercise protocol (i.e., concentric, eccentric) with *post-hoc* test for least significant difference.

Peak values of muscle power in the different tests for positive and negative work were similarly reduced after the eccentric and concentric exercise respective to baseline (Figure 3). For instance, reactive power as assessed on the soft robotic device was reduced by 9% for both the eccentric and concentric protocol. Similarly, Ppeak in the SJs, was 4 and 5% reduced, respectively. Ppeak in the real power test and counter-movement jumps demonstrated reductions only after the eccentric protocol but the magnitude of change was similar to the one seen after the concentric exercise protocol. Similarly, RFD in the reactive force test was reduced after the concentric protocol and showed a trend for a reduction after the eccentric test (*p* = 0.07; Figure 3).

### Metabolic effects of the eccentric and concentric exercise protocol

**Figure 4:**
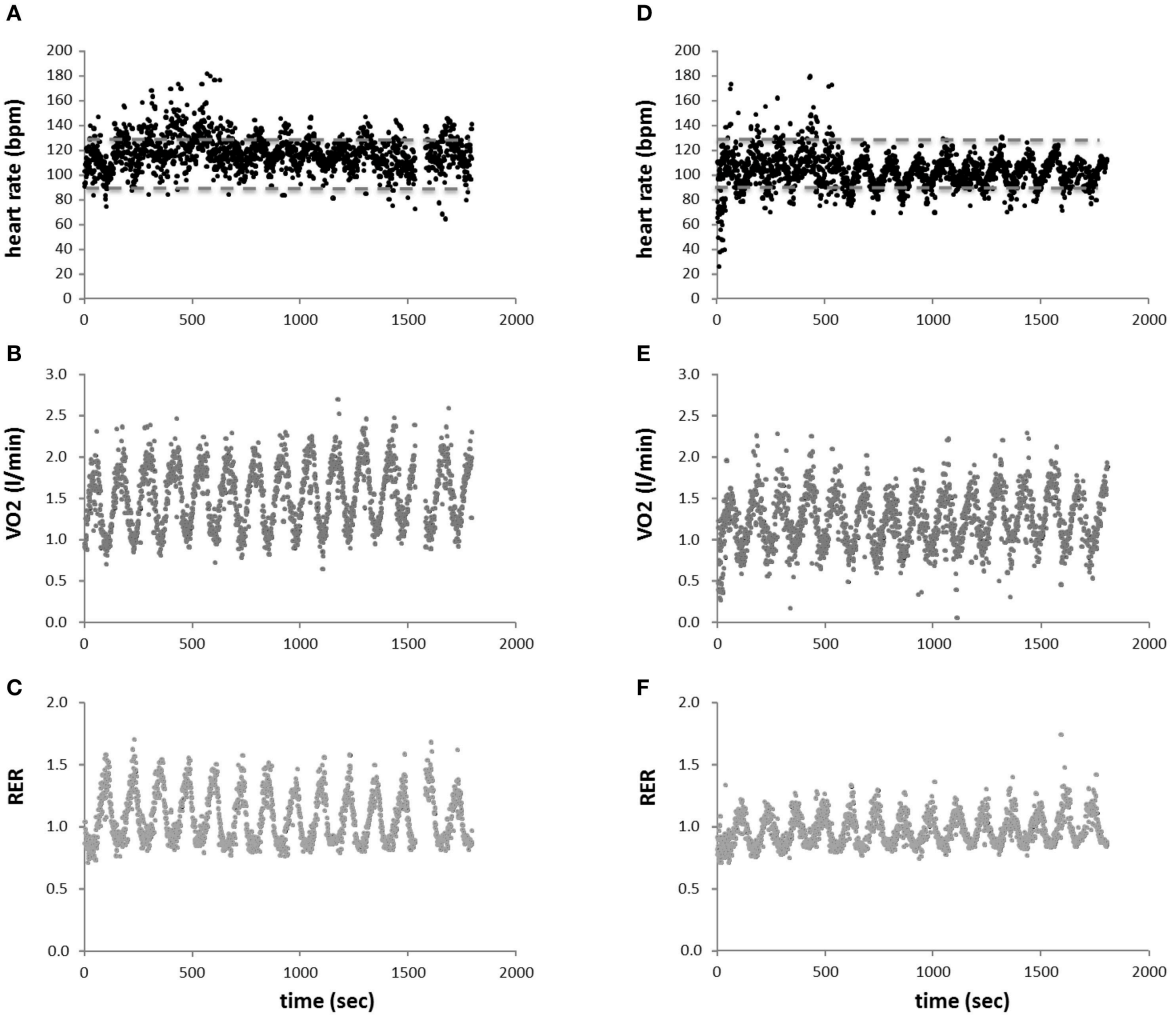
Respiration during interval exercise. Scatter plot of average heart beat **(A,D)**, oxygen uptake **(B,E)**, and respiration exchange ratio (RER) **(C,F)**, over all subjects in function of time over all performed sets of interval-type concentric **(A–C)** and eccentric **(D–F)** exercise. Stippled lines in **(A,D)** indicate upper and lower limits of the interval zone being recommended for the cardiac patient (i.e., 50–70% of peak heart rate). Note the periodic influence of the intervals of exercise and rest.

**Figure 5:**
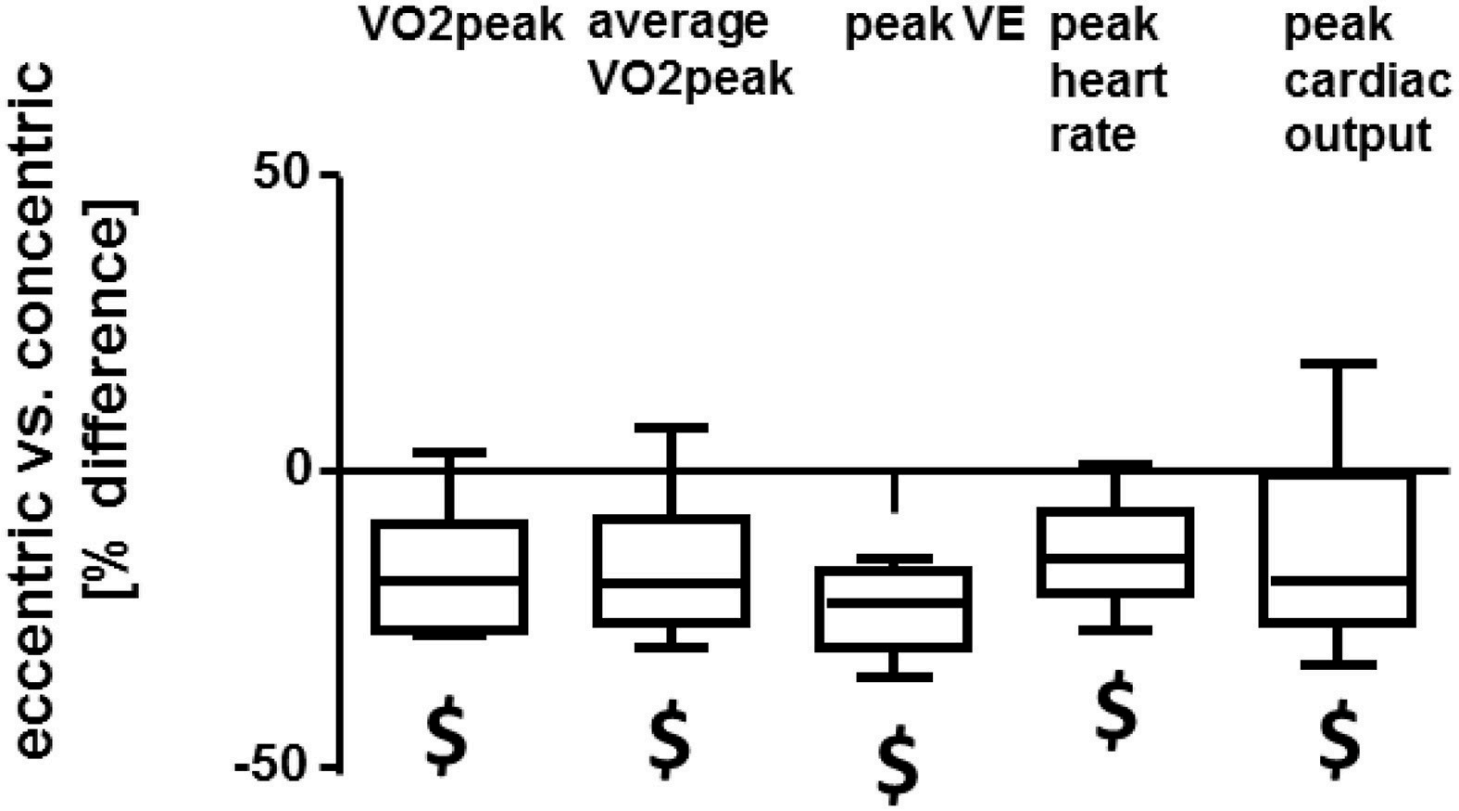
Cardiovascular differences between interval-type concentric and eccentric exercise. Box Whisker plots of the percentage differences between the eccentric vs. the concentric protocol for cardiovascular parameters. $: *p* < 0.05 for eccentric vs. concentric exercise. Repeated ANOVA for the repeated factor of the exercise protocol performed (i.e., concentric, eccentric) with *post-hoc* test for least significant difference.

An example of cardiorespiratory load is given in Figure 4. Heart rate, oxygen and carbon dioxide concentrations varied in a cyclic manner that was paced by the targeted power. Exercise time, average power, and heart rate did not differ between the concentric and eccentric exercise protocol. Cardiovascular parameters of the eccentric exercise differed to those of concentric exercise on the soft robot (Table 2). Figure 5 shows the mean differences between the eccentric and concentric interval exercise for the cardiovascular parameters demonstrating a significant effect of the protocol. Average oxygen uptake (−17%), ventilation (−23%), peak cardiac output (−16%), peak respiration exchange ratio (−13%), and maximal blood lactate (−27%) during soft robotic exercise were lower during the eccentric compared to the concentric exercise.

**Table 2:** Characteristics of the exercise protocols.

**Parameter**	**Eccentric**	**Concentric**	**Constant**	**Ramp**	***p*-value Eccentric vs. concentric**	***p*****-values vs**.		
						**Eccentric**	**Concentric**	**Ramp**
Average power (Watt)	126.71 ± 24.32	125.42 ± 18.81	187.50 ± 37.62	250.00 ± 50.15	0.745	0.004	0.002	0.001
VO_2_ peak (L O2 min^−1^)	**1.85** ± **0.31**	2.22 ± 0.20	2.90 ± 0.45	3.40 ± 0.57	**0.003**	0.001	0.005	0.009
VO_2_ peak per body (mL O2 min-1 kg^−1^)	**24.86** ± **5.37**	26.94 ± 7.52	39.24 ± 5.12	46.05 ± 8.60	**0.011**	0.002	0.002	0.040
Peak VE (L min^−1^)	**60.82** ± **20.51**	79.11 ± 20.93	90.44 ± 19.57	112.79 ± 23.31	**0.001**	0.041	0.454	0.080
Peak cardiac output (L min^−1^)	**11.55** ± **1.02**	13.74 ± 3.00	15.36 ± 4.04	20.80 ± 3.99	**0.043**	0.021	0.009	0.300
Peak heart rate (bpm)	**132.25** ± **23.08**	154.75 ± 24.64	177.63 ± 8.40	181.63 ± 7.95	**0.005**	0.001	0.019	0.300
Average heart rate (bpm)	132.61 ± 29.73	135.15 ± 16.77	161.88 ± 7.61	148.11 ± 10.63	0.785	0.009	0.001	0.001
Average RER	0.86 ± 0.06	0.89 ± 0.11	0.92 ± 0.06	0.91 ± 0.06	0.550	0.020	0.560	0.120
Peak RER	**1.32** ± **0.17**	**1.51** ± **0.20**	1.04 ± 0.08	1.08 ± 0.08	**0.034**	0.002	**0.001**	0.536
Maximal lactate (mM)	**6.15** ± **3.28**	8.46 ± 3.03	7.14 ± 0.82	8.93 ± 2.35	**0.008**	0.366	0.201	0.090
Endtidal pCO2 (mmHg)	30.33 ± 1.87	29.30 ± 1.78	33.38 ± 1.90	32.34 ± 4.38	0.099	0.021	0.010	0.100

*Mean + SD of the metabolic and mechanical characteristics of the soft robotic pedaling exercise under the concentric and eccentric protocol in comparison to ergometer exercise with a constant or incremental (i.e., ramp) load protocol for the eight investigated subjects. P-values are given for the comparison between the eccentric and concentric protocol, and between exercise at constant load and the three other protocols (repeated ANOVA for the factor “protocol” (i.e., eccentric, concentric, constant, or ramp) with post-hoc test of least significant difference). Values, which demonstrate significant differences between the eccentric and concentric types of exercise, are highlighted in bold. For parameter names see the list of abbreviations*.

**Figure 6:**
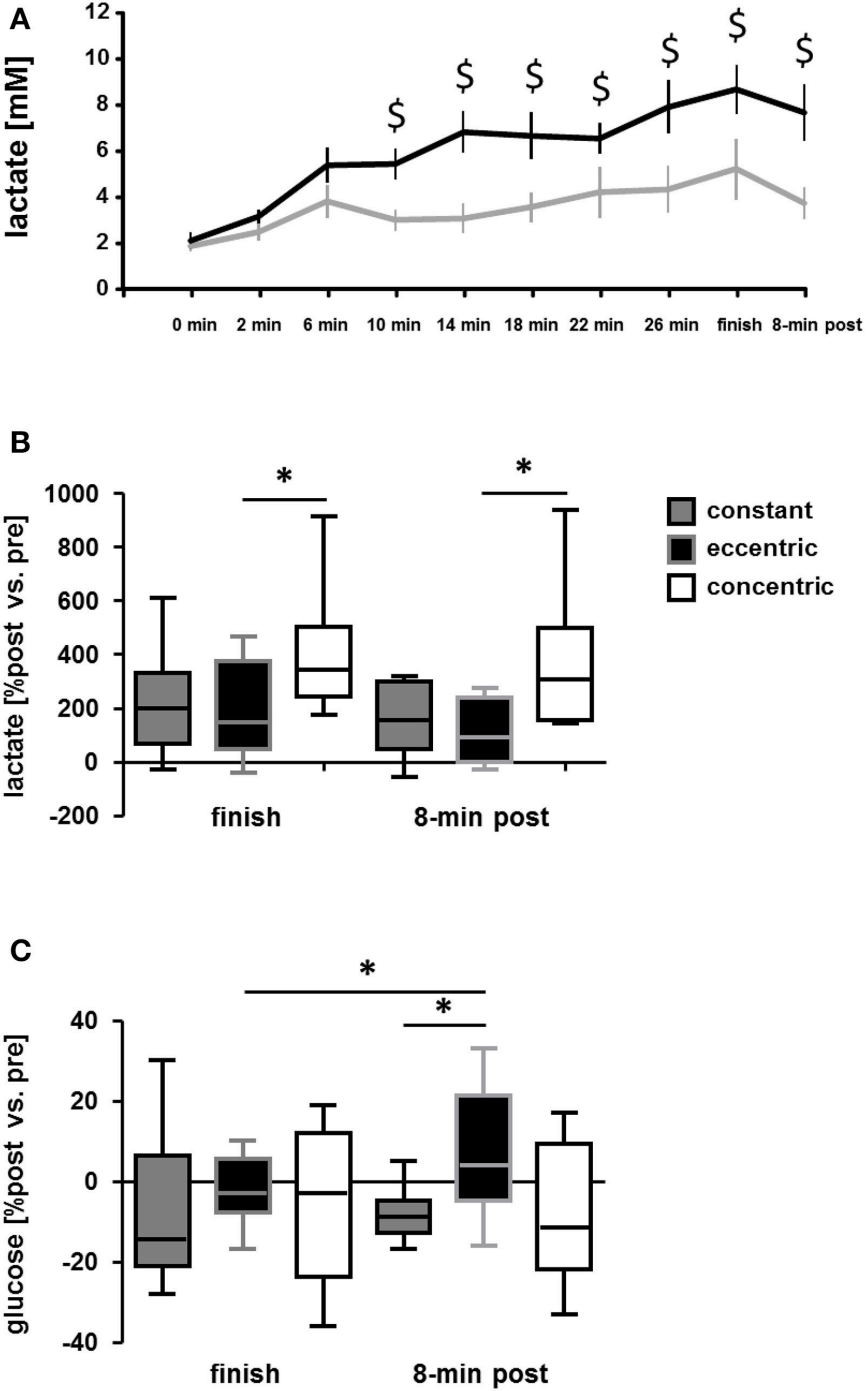
Differences in alterations of serum metabolites between the exercise protocols. **(A)** Line graph showing the mean + SE of blood lactate concentration during the course of matched concentric and eccentric type of interval exercise on the soft robot. **(B,C)** Box Whisker plots showing the baseline-differences in blood lactate **(B)** and glucose **(C)** concentration immediately and 8 after finishing concentric and eccentric interval exercise on the soft robot or endurance exercise at constant load. ^*^p < 0.05 for the indicated comparison. Repeated ANOVA for the repeated factor of the exercise being performed (constant, eccentric, concentric) with *post-hoc* test for least significant difference.

Measurements of blood lactate concentration during exercise on the soft robot demonstrated an effect of time (*p* < 0.001) and interaction effect between time and the exercise protocol (*p* = 0.028). Blood lactate concentrations were increased immediately after exercise with the concentric and the eccentric protocol on the soft robot, and remained increased 8 min after exercise (Figure 6A). The increase in blood lactate concentration was more pronounced with the concentric than the eccentric protocol (Figure 6B). Eight minutes after finishing the eccentric exercise blood glucose concentration was 6% increased respective to the baseline values of 5.3 mM (Figure 6C). Glucose concentration was not affected by concentric exercise.

### Comparing soft robotic to endurance exercise at constant load

The measured peak physiological parameters, except peak VE and maximal lactate, during concentric and eccentric type of exercise on the soft robotic device differed to the values obtained after the constant load type of exercise. VO_2_peak, peak cardiac output, peak heart rate, average heart rate, end tidal CO_2_ all were lower immediately after finishing concentric and eccentric exercise compared to endurance exercise at constant load (Table 2). Similar differences were essentially observed between the interval-type exercise and the ramp protocol during which the peak intensity was higher than during the constant load exercise (Table 2). The intensity of eccentric interval exercise was always below 73% of peak heart rate (Figure 4D). During the concentric interval exercise the intensity was always within 85% of peak heart rate (compare Figure 4D with Figure 4A).

Exercise time (−71%), average power (−33%), and heart rate (−18%) were all lower with the concentric and eccentric interval exercise, respectively, compared to the constant load exercise. By contrast, the rise in blood lactate concentration immediately after finishing the exercise was more pronounced after concentric interval exercise on the soft robot than after constant load exercise on the cycle ergometer (i.e., +7.1 mM vs. 2.8 mM), despite the considerably shorter duration of concentric interval exercise (Figure 6B). The increase in blood glucose concentration 8 min after the eccentric interval exercise (+0.3 mM) respective to baseline exceeded the changes in blood glucose concentration immediately after eccentric exercise (−0.1 mM) and 8-min after constant load exercise (−0.4 mM), respectively; Figure 6C).

### Interrelationships

Network analysis was carried out to identify correlations between those parameters, which were differently affected by eccentric and concentric exercise. Hundred and Sixty-six significant linear relationships were identified, which *r*-value exceeded a value of 0.7. The main theme being reflective of cardiovascular fitness and metabolic strain, i.e., maximal values for VO_2_ and lactate in the constant and incremental tests (Supplementary Table 2). No correlation was seen for values that were assessed during the exercise on the soft robot, except for the number of completed set of exercise during the soft robotic exercise and the RFD measured in the real power test (*r* = −0.76).

For exercise-induced changes, few correlations were identified. Mainly this concerned factors being associated with metabolic strain and the RFD. For instance, negative correlations existed between the fold changes in lactate at finish and 8-min post-exercise with the post- vs. pre- difference in reactive power, i.e., *r* = −0.75 and −0.80, and negative power, i.e., *r* = −0.71, −0.79. As well, blood lactate concentration immediately and 8 min after finishing the eccentric exercise were negatively correlated with the post- vs. pre- difference in the RFD during the negative power test, i.e., *r* = −0.82 and −0.75. Post- vs. pre- differences in force development in the negative power test were correlated with peak RER (P, i.e., *r* = −0.81) and RER when finishing concentric exercise (*r* = −0.71).

## Discussion

The increasing energetic requirements of skeletal muscle physical work vary dependent on the achieved power output and mode of contraction. For instance lengthening type contractions modify the efficiency for the conversion of metabolic energy into mechanical output at any given degree of motor unit recruitment (Bigland-Ritchie and Woods, 1976; Enoka, 1996). We have addressed this contention by assessing the oxygen consumption of eccentric compared to concentric interval-type of leg exercise on identical machinery (i.e., a soft robot) with indirect calorimetry, and compared these values to those of a constant load exercise, which is commonly used in exercise rehabilitation. The aim was to validate the exercise protocols and machinery for a later clinical investigation into rehabilitation of cardiac patients with an interval-type of exercise protocol. The intention was to impose and monitor workload and metabolic alterations in real-time and separately for both legs for interval-type pedaling exercise (https://clinicaltrials.gov/ct2/show/NCT02845063). Toward the end of enhancing the quality and interpretative power of the data, we characterized both power and aerobic capacity of the subjects, as these traits are interdependent (Wilson et al., 2002; Bloxham et al., 2005). Through our investigation we identify that the subjects of our study achieved and completed a same workload per set under the eccentric and concentric exercise protocols (i.e., 7.5 kJ), while indices of cardiorespiratory strain (i.e., peak oxygen uptake, peak ventilation, peak heart rate, and cardiac output; Figure 5), and indices of metabolic strain (i.e., the rise in RER and blood lactate; Table 2, Figures 6A,B), were less pronounced during the eccentric interval-type of exercise compared to the work-matched concentric exercise. As well an overspill of blood glucose was noted after eccentric exercise.

The measured differences after the workout on the soft robot are in line the postulated reduced cardiorespiratory strain with an eccentric contraction protocol compared to an energetically matched concentric protocol (Beaven et al., 2014). Specifically, our findings identify that the reportedly lower metabolic load of continuous cycling eccentric endurance exercise (compared to concentric exercise) also manifests in the setting of an interval-type protocol of pedaling exercise. Based on heart rate interval-type of eccentric exercise lowers cardiorespiratory strain to a level, which is within the 50–70% of peak heart rate, which is the recommended training window for cardiac patients (Figure 4D) (Carre, 2010; Mampuya, 2012). This observation is of specific interest because interval-type exercise provides a potent stimulus at a reduced net time of exercise (Helgerud et al., 2007; Hood et al., 2011; Fu et al., 2014). The suitability of the deployed interval-type of eccentric exercise on the soft robot for the cardiac patient is supported from the measured cardiac output of 11.5 L min^−1^, which corresponds to 56% of the peak values (Table 2).

The mechanisms involved in the reduced oxygen consumption of eccentric contractions compared to work-matched concentric contractions have been described before to include distinct muscular and neuronal processes. For instance, a higher efficiency has been described for the cross bridge cycle and elastically stored energy is released from the muscle-tendon unit when muscle contracts eccentrically (Ryschon et al., 1997; Fang et al., 2004; Isner-Horobeti et al., 2013; Nishikawa, 2016). The former two energetic characteristics, both, reduce the necessity for energy provision during muscle contraction. Here we quantify the contribution of metabolic processes reflecting respiration and glucose metabolism to be an approximate of 15% lower with eccentric compared to work- concentric contractions (Table 2, Figure 5). Our results show that glucose concentration in blood is elevated immediately after eccentric exercise. This relates to the reported impairment of glucose import and glucose metabolism after eccentric exercise (Kirwan et al., 1992; Asp et al., 1995; Kristiansen et al., 1996; Kirwan and del Aguila, 2003; Philippe et al., 2016); especially as glucose is increasingly released from hepatic sources into the blood stream with exercise and imported via capillary perfusion into contracting muscle fibers (Wahren et al., 1975; Clifford and Hellsten, 2004). Interestingly, no differences were observed in the post- vs. pre- reductions for peak values of real power (*p* = 0.691), reactive power (*p* = 0.864), and negative power (*p* = 0.416) post-exercise between the eccentric and concentric protocol (Figure 3). Measured values of power reduction post-exercise were also not correlated to the alterations in blood glucose. These observations in physically active men suggest that the reduction of glucose import after eccentric interval-type exercise on the soft robot may not be primarily related to muscle fatigue.

A lower peak respiration exchange ratio during interval-type eccentric exercise compared to concentric exercise, i.e., values of 1.32 vs. 1.51, indicates that glucose metabolization was lower during the eccentric exercise. This difference corresponded to an ~15% lower cardiorespiratory strain based on oxygen uptake and peak cardiac output. By contrast the total work produced during the two types of soft robot exercise did not differ and amounted to 7.5 ± 0.1 kJ per set for both the concentric and eccentric protocol. This indicates that differences in the cardiovascular cost of eccentric and concentric exercise with the deployed interval-type protocol on the soft robot are explained by a reduced aerobic combustion of glucose-derived metabolites. This mechanism is supported by the distinctly lower degree of increase in blood lactate during and after eccentric exercise compared to concentric exercise (Figures 6A,B), as this metabolite indicates an insufficient aerobic combustion of pyruvate during elevated glycolysis (reviewed by Schmutz et al., 2010). The higher work efficiency of eccentric contractions therefore possibly also reflects a lower degree of fiber recruitment during eccentric compared to work-matched concentric exercise (reviewed in Duchateau and Enoka, 2016). Because perfusion of capillaries is dictated by the contraction of muscle fibers which situate in their neighborhood, the increased glucose concentration with our exercise protocol may be due to a reduced degree of capillary recruitment to the perfused vascular network in contracting muscle (Clifford and Hellsten, 2004).

The specific effects of interval-type eccentric exercise on the proxy of metabolic strain, blood lactate, compare well to the reportedly lower rises in blood lactate after work-matched short intense or endurance type eccentric exercise on an isokinetic dynamometer/eccentric bike (Bonde-Petersen et al., 1972; Horstmann et al., 2001; Penailillo et al., 2013). Conversely, fold changes in the concentration of blood lactate, and respiration exchange ratio, were negatively co-related to changes in RFD in the reactive and negative power test. This observation is in line with the expectation where a higher glucose utilization during exercise, as visualized in larger increases in blood lactate and RER (Sahlin et al., 1987), would lead to an enlarger decrease in muscles functional ability to perform work. Because external work was matched in our study, we interpret our findings to indicate that differences in both import and aerobic respiration of glucose in skeletal muscle contribute to the metabolic differences between eccentric and concentric exercise.

The combination of soft robotic exercise and ergospirometry allowed to control and monitor the achieved workload, including the implicated type of contractions, and to measure the associated oxygen consumption, contraction-by-contraction, and breath-by-breath. This permitted to quantify mechanical and metabolic differences between eccentric and concentric exercise and pinpoint to the contribution of muscular and cardiovascular mechanisms. The technical options of the soft robot also enabled drawing preliminary conclusions of the failure of certain subjects to complete the requested 15 sets of 30 cycles of two-leg knee extension and flexion. For instance, *post-hoc* evaluation of the data revealed that for the three subjects, whom finished the concentric exercise session prematurely, performance values fell below 87% of the target power in the last set of contractions, thus necessitating further adjustments in the number of sets to be performed in the subsequent eccentric protocol to match both workload of interval exercise. The inspection of power development during the soft robotic tests also showed higher values of RFD for the three subjects finishing the exercise prematurely (i.e., mean values of 2,420 vs. 1,668 N s^−1^, *p* = 0.01). As well a correlation existed between RFD in the real power test and the effectively completed number of sets (*r* = −0.76). The results indicate that the failure to complete the target exercise is related to a faster production of the target power. A number of biological factors, including the improved coordination between visual input and motor recruitment, and muscle fiber type composition, may explain this observation (Jones et al., 2004).

A conspicuous observation of our investigation was reflected in the fact that a considerable portion of the power was produced in the “off-phase” of the concentric protocol, when the leg is subject to knee flexion. In this regard, it is of interest that the evaluation of the developed force in a work cycle showed that the requested mix of eccentric and concentric movement was not always met (Figure 2). This “extra” eccentric work during the concentric protocol possibly reflects that the soft robotic exercise was performed in a closed kinetic chain where subjects were asked to always press against uncoupled pedals. Thus, forward movement of the pedal, i.e., against the body of the subjects, solicits a counter-movement of the involved leg to slow the pedal moving toward the body, especially as subjects were instructed to always press against the pedal to hold the position of the foot respective to the pedal. Possibly subjects were unable to relax (or de-recruit) the involved knee extensor muscles sufficiently rapid to comply to the task. This circumstance is indicated by the moderate rise in (normalized) force during the work cycle with the transition from knee extension to flexion during the concentric protocol (Figure 2C). This results in extra eccentric work being performed during the concentric protocol. Similarly, the de-recruitment required to avoid the production of positive power in the extension phase of the eccentric protocol, when the pedal moves away from the body, may not be achieved by all the subjects. Notably the extra work during the interval-types of exercise appeared despite that visual feedback was provided to the subjects to match their performance respective to the target power.

The advantage of eccentric types of exercise for subjects with cardiovascular limitations has been discussed before (Rooyackers et al., 2003; Philippe et al., 2016). Eccentric exercise is also suited for subjects wishing to pronounce gains in maximal muscle force (i.e., muscle strength; (Farthing and Chilibeck, 2003). This effect has been shown to relate to specific muscle adaptations. For instance it has been shown that eccentric forms of endurance exercise result in a stronger stimulus for muscle hypertrophy and capillary growth than comparable concentric exercise (LaStayo et al., 2000). Continuous forms of exercise may be metabolically too challenging for subjects with a deconditioned heart, which could especially benefit from improved muscle capillarization as this counteracts the development of hypertension, type II diabetes and other aspects of the metabolic syndrome (Cornelissen and Smart, 2013). In this perspective, we devised our protocol to incorporate an interval-type stimulus, which allows for recovery between sets of exercise. The previously used eccentric bikes did not to constitute a viable option for our investigation because they are only custom manufactured (LaStayo et al., 2000; Zoll et al., 2006; Dufour et al., 2007) and require intensive individual technical support (personal communication). We therefore performed our investigation on a soft robot, which is commercially produced and did allow improved control over the straining stimulus. The intention of our approach was to identify whether mechanical tests on the soft robot to quantify real, negative, or reactive power during single leg extensions would provide reference values to set power output for the interval protocol, determine functional effects of eccentric exercise, and to compare how these values relate to a standard measure being used to determine power output of single contractions, i.e., vertical jumps. Our results support the usefulness of the soft robot-based power measurements. However, based on the ratio between the developed positive to negative power, and the finding that three subjects with the highest RFDs failed to complete 15 sets of concentric exercise, it appears that the deployed interval-type protocol on the soft robot requires refinement. In this regard, the negative correlation between the RFD during repeated counter-movement jumps and the sets performed is of interest. This result indicates the possibility to apply the test measures of mechanical performance on the soft robot to tailor the interval-type of exercise to the individual mechanical and metabolic capacities of a subject. Improvement may be provided by a mechanically less demanding protocol that is personalized respective to the individual capacity. This may comprise software adjustments as well, which tailor the rate during which force need to be developed according to the individual capacities. For instance, RFD during squat and vertical jumps is known to be considerably lower in normal physically active subjects compared to volley ball players (McLellan et al., 2011; Haff et al., 2015), falling below the values 15 kN s^−1^ at which the PAMs operate. As well the target work load may be set lower than the 17% of maximal power in the real power test, which is based on a single contraction. Alternatively, reference values may be used to set the intensity of an endurance stimulus to values between 40 and 50% of maximal oxygen uptake (Gordon et al., 2004). This would require however the additional measure of this parameter in a ramp test to exhaustion but which is time consuming and not routinely available in the setting of a fitness or rehabilitation center. The failure to maintain the requested power output may also be explained by the specific requirements on motor control and coordination between the independently operating legs during the pedaling on the robotic device. The exercise may also lead to obstruction of blood flow (Heyward and McCreary, 1978) and metabolic fatigue due to the relatively high load on extensor muscles of both legs. Such a problem would be reduced with a protocol that is more reflective of the movement pattern of bicycle exercise where the two connected pedals allow a phasic control of muscle contraction and relaxation (recruitment and de-recruitment respectively).

The measures on the eight subjects which completed he entire protocol, allowed to identify a number of expected effects, i.e., lower VO_2_peak, average VO_2_, peak heart rate, peak cardiac output during eccentric compared to work-matched concentric pedaling exercise (Beaven et al., 2014), and a mitigated increase in blood lactate concentration and increased blood glucose concentration respective to baseline in response to eccentric compared to concentric pedaling exercise. Our study was however underpowered to identify a significant difference between the eccentric and concentric exercise protocol for the effective, not-baseline related blood glucose concentration. For this difference to resolve at the level of statistical significance, a prospective number of 12 subjects would have been required (Supplementary Table 1). Meanwhile the power analysis predicts, that exercise protocol-specific changes in muscle's mechanical characteristics post-exercise would have revealed in the negative power tests with a doubling of the subject number.

It may be considered a limitation that due to a missing randomization no cross-over design was adopted in our investigation, i.e., that the concentric exercise session was always carried out prior to the eccentric exercise. However, we assume that a carry-over effect of the sequence of the exercise sessions on the assessed parameters can be reasonably excluded due to the long rest period between the exercise sessions (i.e., 1 week), and the fact that familiarizations were carried. However, as we did not collect data being directly related to muscle damage, we cannot validate this contention. For instance it is understood from the literature that one bout of concentric exercise as carried out in our investigation does not affect the molecular and systemic metabolic exercise response for more than 24–48 h (e.g., Mikines et al., 1988; Egan and Zierath, 2013). Inversing the order of exercise (in a cross-over design) was not an option because eccentric exercise specifically affects homeostasis in skeletal muscle such as performance, muscle water content and volume, and glucose metabolism in possible relationship to strain induced muscle damage (Kirwan et al., 1992; Foley et al., 1999). By contrast the incorporation of a cross over design with prior eccentric exercise would have added the influence of muscle damage at the selected time point 1 week after the first exercise session in week 4 (Friden et al., 1983; Child et al., 1998).

Also, the absence of measurements of blood pressure and additional measurements post-exercise to accurately determine peak lactate values may be considered a limitation; especially as the latter parameter is a recommended guide to set exercise intensity (Goodwin et al., 2007). The lack of measures on blood pressure does not allow to fully apprehend cardiovascular stress during exercise, which has been shown to be lower with eccentric exercise (Overend et al., 2000; Meyer et al., 2003). We note that the selected time point of measure 8 min post-exercise, is in line with the recommended window (Goodwin et al., 2007) and that the measured values for maximal blood lactate concentration corroborate the conclusion from the other characterized parameters, such as cardiac output, VO_2_peak, peak VE, and heart rate, on the lower metabolic cost of eccentric compared to concentric exercise. Especially the in here quantified cardiac output, which is an integrated measure of cardiovascular work, adds important information a lower demand for tissue perfusion during the employed eccentric exercise protocol.

## Conclusion

The interval-type of eccentric exercise being established on the soft robot allows unloading the heart and reduces metabolic strain to contracting muscles, despite a considerable mechanical output, and produces larger increases in glucose handling. The deployed protocol needs however to be revisited because considerable extra negative and positive work was produced with the imposed closed chain concentric and eccentric exercise, respectively, and because the protocol was too demanding for some of the healthy subjects of our study, although the intensity of exercise situated within the recommendations for subjects with cardiac limitations. In this regard, a personalized setting for the rate at which maximal power needs be developed is indicated. Mechanical measurements on force development in the dynamic situation of endurance exercise on the soft robot may be used to tailor the target load during exercise to the individual biological capacity.

## Ethics statement

This study was carried out in accordance with the recommendations of Ethics Commission of the Canton of Zurich, Switzerland with written informed consent from all subjects. All subjects gave written informed consent in accordance with the Declaration of Helsinki. The protocol was approved by the Ethics Commission of the Canton of Zurich, Switzerland.

## Author contributions

MF and ML contributed to conception and design of research; MF, RB, and ML analyzed data; MF, RB, and ML interpreted results of experiments; MF organized funding; MF, ML prepared figures; MF, RB, drafted the manuscript. All authors approved the manuscript.

### Conflict of interest statement

ML is Chief Technology Officer of Dynamic Devices that assembles the tested soft robot. The other authors declare that the research was conducted in the absence of any commercial or financial relationships that could be construed as a potential conflict of interest.

## Abbreviations

arc_len: arc length
CMJ: counter-movement-jump
kN: kilo Newton
PAM: pneumatic artificial muscles
MJ1L: one-legged multiple jumps
ms: milli seconds
N: Newton
Ppeak: peak power
Pcmjpeak: peak performance CMJ
Ppeakneg: peak performance negative power test
Ppeakramp: peak performance ramp test
Ppeakreac: peak performance reactive power test
Ppeakreac: peak performance real power test
Psjpeak: peak performance SJ
RER: respiration exchange ratio
RFD: rate of force development
s: seconds
SE: Standard error
SJ: squat-jump
VE: ventilation
VO_2_: oxygen uptake
VO_2_peak: peak oxygen uptake in the ramp test
W: Watt
