# Supplementary material for: Cardiovascular and Muscular Consequences of Work-Matched Interval-Type of Concentric and Eccentric Pedaling Exercise on a Soft Robot
Source: Front Physiol. 2017 Aug 31;8:640. doi: 10.3389/fphys.2017.00640 (PMC5583980; doi:10.3389/fphys.2017.00640)
Supplement: Supplementary file 1 [file Table1.DOCX]

***Supplementary table 1: Summary of the post-hoc calculations in the observed effect sizes and power in the 8 studied subjects.***

P-values, effect size (partial eta square) and power was determined using repeated measures ANOVA (SPSS) from the effective values. The prospective number of samples (n) to reach statistical significance were calculated with G-power (version 3.1.9.2, Faul Universität Kiel, Germany) based on the effect sizes.

Cardiovascular and muscular consequences of work-matched interval-type of concentric and eccentric pedalling exercise on a soft robot

Martin Flück*, Rebekka Bosshard, Max Lungarella

* Correspondence: Martin Flück: e-mail: mflueck@research.balgrist.ch

*Repeated ANOVA for the repeated factors ‘protocols’ (ECC x CON x constant) and ‘time’ (0-min, immediately post, 8-min post)*

***parameter factor p-value effect size power prospective n***

blood lactate concentration protocol 0.001 0.661 0.991 6

blood lactate concentration time <0.001 0.750 1.000 6

blood lactate concentration protocol x time 0.001 0.505 0.986 8

blood lactate concentration vs. 0-min protocol 0.074 0.581 0.501 9

blood lactate concentration vs. 0-min time 0.018 0.573 0.749 6

blood lactate concentration vs. 0-min protocol x time 0.859 0.050 0.065 28

blood glucose concentration protocol 0.522 0.089 0.142 48

blood glucose concentration time 0.196 0.208 0.318 27

blood glucose concentration protocol x time 0.375 0.136 0.301 12

blood glucose concentration vs. 0-min protocol 0.317 0.369 0.192 68

blood glucose concentration vs. 0-min time 0.548 0.063 0.084 84

blood glucose concentration vs. 0-min protocol x time 0.044 0.713 0.627 8

*Repeated ANOVA for the repeated factor ‘protocols’ (ECC x CON)*

***parameter factor p-value effect size power prospective n***

average power protocol 0.745 0.128 0.142 34

VO2 peak protocol 0.003 0.734 0.963 6

VO2peakM protocol 0.011 0.700 0.931 6

averageVO2peak protocol 0.016 0.644 0.784 6

peak VE protocol 0.001 0.903 1.000 4

peak cardiac output protocol 0.043 0.467 0.568 8

peak heart rate protocol 0.005 0.703 0.934 6

average heart rate protocol 0.785 0.011 0.057 368

average RER protocol 0.550 0.054 0.085 80

peak RER protocol 0.034 0.503 0.628 8

peak lactate protocol 0.008 0.660 0.883 6

endtidal CO2 protocol 0.099 0.341 0.377 12

*Repeated ANOVA for the repeated factors ‘protocols’ (ECC x CON) and ‘time’ (pre, post)*

***parameter factor p-value effect size power prospective n***

reactive power protocol 0.104 0.332 0.365 12

reactive power time 0.001 0.791 0.992 6

reactive power protocol x time 0.997 0.000 0.050 3340

RFD reactive power test protocol 0.153 0.268 0.283 16

RFD reactive power test time 0.001 0.828 0.998 4

RFD reactive power test protocol x time 0.818 0.008 0.055 168

real power protocol 0.725 0.019 0.062 228

real power time 0.022 0.553 0.714 8

real power protocol x time 0.702 0.022 0.064 64

RFD real power test protocol 0.210 0.214 0.224 20

RFD real power test time 0.207 0.216 0.226 20

RFD real power test protocol x time 0.480 0.074 0.099 20

negative power protocol 0.002 0.830 0.993 4

negative power time 0.399 0.121 0.120 36

negative power protocol x time 0.415 0.113 0.115 16

RFD negative power test protocol 0.515 0.074 0.090 60

RFD negative power test time 0.409 0.116 0.117 38

RFD negative power test protocol x time 0.376 0.132 0.128 12

Power CMJ protocol 0.455 0.096 0.104 44

Power CMJ time 0.039 0.535 0.595 8

Power CMJ protocol x time 0.894 0.003 0.052 540

Power SJ protocol 0.383 0.129 0.126 34

Power SJ time 0.014 0.666 0.819 6

Power SJ protocol x time 0.408 0.116 0.117 16

Power MJ1L protocol 0.092 0.401 0.393 10

Power MJ1L time 0.992 0.000 0.050 11040

Power MJ1L protocol x time 0.979 0.000 0.050 3340
