# Supplementary material for: Cardiovascular and Muscular Consequences of Work-Matched Interval-Type of Concentric and Eccentric Pedaling Exercise on a Soft Robot
Source: Front Physiol. 2017 Aug 31;8:640. doi: 10.3389/fphys.2017.00640 (PMC5583980; doi:10.3389/fphys.2017.00640)
Supplement: Supplementary file 2 [file Table2.DOCX]

***Supplementary table 2: Interrelationships between parameters***

Cardiovascular and muscular consequences of work-matched interval-type of concentric and eccentric pedalling exercise on a soft robot

Martin Flück*, Rebekka Bosshard, Max Lungarella

* Correspondence: Martin Flück: e-mail: mflueck@research.balgrist.ch

List of parameters which demonstrate significant correlations at an r-value > 0.7.

***parameter 1 parameter 2 r-value***

ConstGlucA IncVE0 -0.86

ConstHR0 CritPHR0 0.83

ConstHR10 ConstHR0 0.85

ConstHR10 ConstHR2 0.74

ConstHR10 ConstHR4 0.90

ConstHR10 ConstHR6 0.95

ConstHR10 ConstHR8 0.96

ConstHR12 ConstHR0 0.82

ConstHR12 ConstHR10 0.96

ConstHR12 ConstHR2 0.78

ConstHR12 ConstHR4 0.89

ConstHR12 ConstHR6 0.95

ConstHR12 ConstHR8 0.97

ConstHR14 ConstHR0 0.72

ConstHR14 ConstHR10 0.96

ConstHR14 ConstHR12 0.94

ConstHR14 ConstHR2 0.79

ConstHR14 ConstHR4 0.95

ConstHR14 ConstHR6 0.97

ConstHR14 ConstHR8 0.94

ConstHR16 ConstHR0 0.83

ConstHR16 ConstHR10 0.97

ConstHR16 ConstHR12 0.95

ConstHR16 ConstHR14 0.95

ConstHR16 ConstHR2 0.73

ConstHR16 ConstHR4 0.90

ConstHR16 ConstHR6 0.94

ConstHR16 ConstHR8 0.93

ConstHR18 ConstHR0 0.78

ConstHR18 ConstHR10 0.98

ConstHR18 ConstHR12 0.96

ConstHR18 ConstHR14 0.98

ConstHR18 ConstHR16 0.98

ConstHR18 ConstHR2 0.72

ConstHR18 ConstHR4 0.92

ConstHR18 ConstHR6 0.94

ConstHR18 ConstHR8 0.95

ConstHR4 ConstHR2 0.85

ConstHR6 ConstHR0 0.77

ConstHR6 ConstHR2 0.87

ConstHR6 ConstHR4 0.95

ConstHR6 IncGluc0 0.71

ConstHR8 ConstHR0 0.79

ConstHR8 ConstHR2 0.83

ConstHR8 ConstHR4 0.93

ConstHR8 ConstHR6 0.96

ConstHRA CritPHR8n 0.76

ConstHRA IncHR8n 0.73

ConstHRA IncHRA 0.77

ConstHRA IncHRpeak 0.78

ConstHRpeak ConstHR10 0.70

ConstHRpeak ConstHR14 0.74

ConstHRpeak ConstHR16 0.70

ConstHRpeak ConstHR18 0.72

ConstHRpeak ConstHR4 0.78

ConstHRpeak ConstHR6 0.74

ConstHRpeak ConstHRA 0.97

ConstHRpeak CritPHR8n 0.72

ConstHRpeak IncHRA 0.70

ConstHRpeak IncHRpeak 0.71

ConstLac0 IncLac0 -0.77

ConstLac0 IncLac2 -0.70

ConstLac0 IncVE0 -0.74

ConstLac14 ConstLac10 0.90

ConstLac14 IncRER4 0.73

ConstLac2 IncRERpeak 0.78

ConstLac2 IncVO2Mpeak 0.72

ConstLac2 IncVO2peak 0.73

ConstLac6 IncRER4 0.73

ConstLac6 IncRER6 0.73

CritPCritP CritPHRA 0.74

CritPCritP CritPHRpeak 0.72

CritPHR2 CritPHR1 0.96

CritPHRA CritPHR1 0.86

CritPHRA CritPHR2 0.92

CritPHRpeak CritPHR1 0.87

CritPHRpeak CritPHR2 0.93

CritPHRpeak CritPHRA 1.00

IncAF2 IncHR8n -0.73

IncAF4 IncAF2 0.91

IncAF6 IncAF2 0.81

IncAF6 IncAF4 0.92

IncAF6 IncVE6 0.72

IncAFpeak IncAFA 0.96

IncGluc0 CritPHR0 0.77

IncHR0 CritPHR1 0.76

IncHR0 CritPHR2 0.81

IncHR0 CritPHR8n 0.86

IncHR0 CritPHRA 0.76

IncHR0 CritPHRpeak 0.77

IncHR2 CritPHR8n 0.71

IncHR2 IncHR0 0.81

IncHR4 IncHR0 0.74

IncHR4 IncHR2 0.96

IncHR6 CritPHR8n 0.71

IncHR6 IncHR0 0.73

IncHR6 IncHR2 0.95

IncHR6 IncHR4 0.98

IncHR8n IncHR2 0.70

IncHR8n IncHRA 0.75

IncHRpeak IncHR8n 0.75

IncHRpeak IncHRA 1.00

IncLac0 CritPCritP 0.78

IncLac0 CritPHRA 0.73

IncLac0 CritPHRpeak 0.71

IncLac2 CritPLac8n 0.71

IncLac2 IncLac0 0.78

IncLac6 IncLac2 0.85

IncLac8n CritPHR8n 0.76

IncRER0 IncLac2 0.76

IncRER2 CritPCritP -0.72

IncRER2 IncGluc8n -0.76

IncRER4 IncRER2 0.89

IncRER6 CritPCritP -0.71

IncRER6 CritPLac8n -0.71

IncRER6 IncRER2 0.80

IncRER6 IncRER4 0.91

IncRERpeak IncHR8n 0.71

IncRERpeak IncRERA 0.82

IncVE4 IncVE2 0.72

IncVE6 IncVE4 0.91

IncVEpeak IncVEA 0.91

IncVO20 CritPCritP 0.81

IncVO20 CritPLacA 0.75

IncVO20 IncLac0 0.83

IncVO22 CritPLac8n 0.84

IncVO24 IncRER6 -0.71

IncVO24 IncVO22 0.82

IncVO2A IncVEA 0.79

IncVO2A IncVEpeak 0.72

IncVO2M0 CritPCritP 0.74

IncVO2M0 CritPLac8n 0.80

IncVO2M0 IncLac0 0.78

IncVO2M0 IncLac2 0.71

IncVO2M0 IncRER0 0.72

IncVO2M0 IncVO20 0.90

IncVO2M2 CritPLac8n 0.76

IncVO2M2 IncVO22 0.74

IncVO2M2 IncVO2M0 0.80

IncVO2M4 IncVEA 0.74

IncVO2M4 IncVO2A 0.78

IncVO2M4 IncVO2M0 0.82

IncVO2M4 IncVO2M2 0.97

IncVO2M4 IncVO2peak 0.74

IncVO2M6 IncVEA 0.70

IncVO2M6 IncVO2A 0.72

IncVO2M6 IncVO2M0 0.81

IncVO2M6 IncVO2M2 0.96

IncVO2M6 IncVO2M4 0.98

IncVO2MA IncVEA 0.77

IncVO2MA IncVO2A 0.94

IncVO2MA IncVO2M2 0.86

IncVO2MA IncVO2M4 0.93

IncVO2MA IncVO2M6 0.90

IncVO2MA IncVO2peak 0.86

IncVO2Mpeak IncVEA 0.78

IncVO2Mpeak IncVEpeak 0.78

IncVO2Mpeak IncVO2A 0.91

IncVO2Mpeak IncVO2M2 0.86

IncVO2Mpeak IncVO2M4 0.92

IncVO2Mpeak IncVO2M6 0.89

IncVO2Mpeak IncVO2MA 0.98

IncVO2Mpeak IncVO2peak 0.92

IncVO2peak IncVEA 0.80

IncVO2peak IncVEpeak 0.87

IncVO2peak IncVO2A 0.91
