# Supplementary material for: Cardiovascular and Muscular Consequences of Work-Matched Interval-Type of Concentric and Eccentric Pedaling Exercise on a Soft Robot
Source: Front Physiol. 2017 Aug 31;8:640. doi: 10.3389/fphys.2017.00640 (PMC5583980; doi:10.3389/fphys.2017.00640)
Supplement: Supplementary file 3 [file Image1.PDF]

Supplementary figure 1

Cardiovascular and muscular consequences of work-matched interval-type of concentric and eccentric pedalling exercise on a soft robot

Martin Flück*, Rebekka Bosshard, Max Lungarella

*** Correspondence:** Martin Flück: e-mail: mflueck@research.balgrist.ch

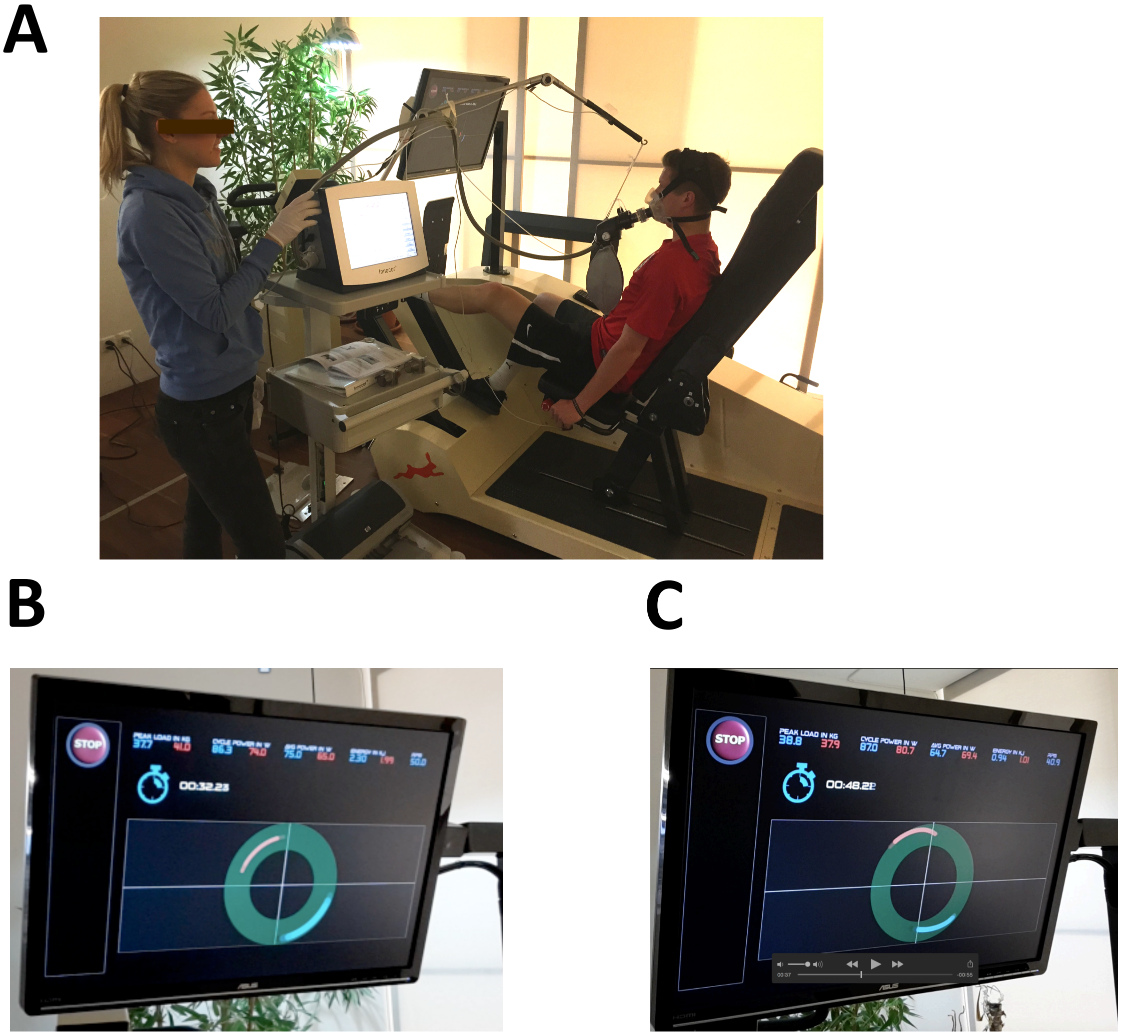

Supplementary figure 1. Overview of the soft robotic device. A) Lateral view of the Allegro soft robotic device during the instruction of a subject. B, C) Screen shots illustrating the visual feedback for the deployed inertia wheel protocol. The shots provide examples each of a case where the subject performs in the target zone (B) or slightly outside the green target zone being indicated by the green-coloured ring on the screen (C). Blue and red stripes symbolize the left and right leg.
