# Supplementary material for: Cardiovascular and Muscular Consequences of Work-Matched Interval-Type of Concentric and Eccentric Pedaling Exercise on a Soft Robot
Source: Front Physiol. 2017 Aug 31;8:640. doi: 10.3389/fphys.2017.00640 (PMC5583980; doi:10.3389/fphys.2017.00640)
Supplement: Supplementary file 4 [file Image2.PDF]

Supplementary figure 2

Cardiovascular and muscular consequences of work-matched interval-type of concentric and eccentric pedalling exercise on a soft robot

Martin Flück*, Rebekka Bosshard, Max Lungarella

*** Correspondence:** Martin Flück: e-mail: mflueck@research.balgrist.ch

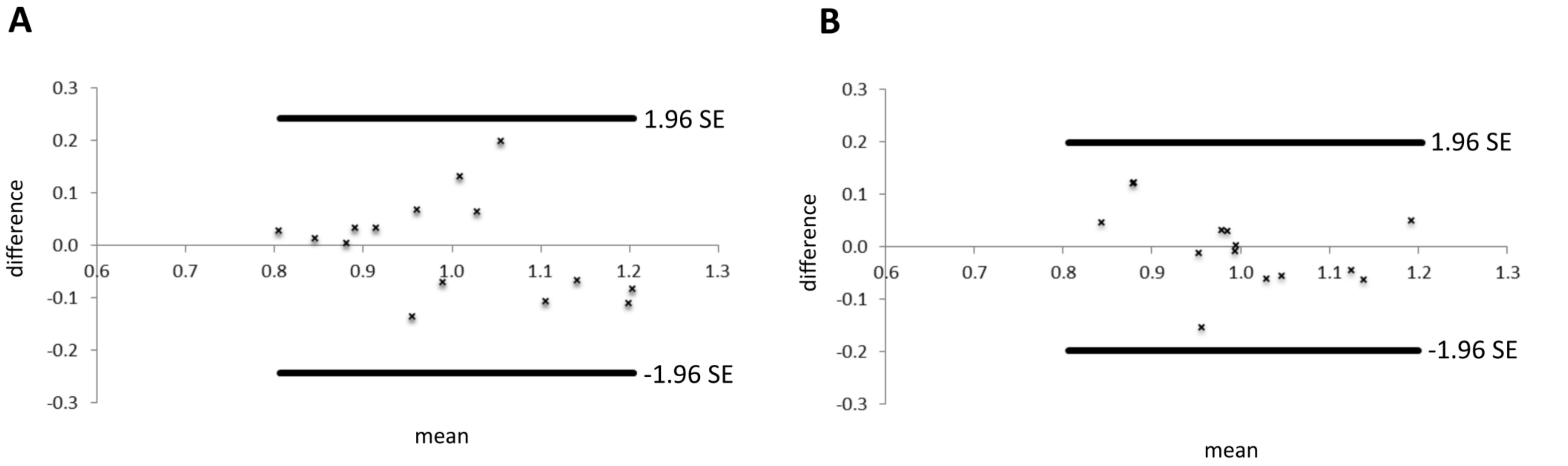

Supplementary figure 2. Bland Altmann plots comparing the mean versus the difference of normalized values for power (A) and force (B) between the soft robotic measurements for real power and mechanographic measurements of the squat jumps. Crosses reflect the individual values and lines indicate the agreement limits (i.e. 1.96 SE).
